# Five new cases of syndromic intellectual disability due to *KAT6A* mutations: widening the molecular and clinical spectrum

**DOI:** 10.1186/s13023-020-1317-9

**Published:** 2020-02-10

**Authors:** Roser Urreizti, Estrella Lopez-Martin, Antonio Martinez-Monseny, Montse Pujadas, Laura Castilla-Vallmanya, Luis Alberto Pérez-Jurado, Mercedes Serrano, Daniel Natera-de Benito, Beatriz Martínez-Delgado, Manuel Posada-de-la-Paz, Javier Alonso, Purificación Marin-Reina, Mar O’Callaghan, Daniel Grinberg, Eva Bermejo-Sánchez, Susanna Balcells

**Affiliations:** 1 Department of Genetics, Microbiology and Statistics, Faculty of Biology, University of Barcelona, IBUB, IRSJD, Barcelona, Spain; 2 Centro de Investigaciones Biomédicas en Red de Enfermedades Raras (CIBERER), Instituto de Salud Carlos III (ISCIII), Madrid, Spain; 3 Present address: Neurometabolic Unit, Hospital Sant Joan de Déu, Barcelona, Spain; 4 Institute of Rare Diseases Research (IIER), Instituto de Salud Carlos III (ISCIII), Madrid, Spain; 5 Department of Genetic and Molecular Medicine and Pediatric Rare Diseases Institute (IPER), Institut de Recerca Sant Joan de Déu (IRSJD), Hospital Sant Joan de Déu, Barcelona, Spain; 6 Genetics Unit, University Pompeu Fabra, Hospital del Mar Research Institute IMIM, Barcelona, Spain; 7 Women’s and Children’s Hospital, South Australian Health and Medical Research Institute and The University of Adelaide, Adelaide, Australia; 8 Department of Neurology, Hospital Sant Joan de Déu, Barcelona, Spain; 9 Dysmorpholgy and Clinical Genetics, Division of Neonatology, Neonatal Research Unit, Hospital Universitario y Politécnico La Fe, Valencia, Spain

**Keywords:** KAT6A, Neurodevelopmental disease, Clinical genetics, Whole exome sequencing, Clinical characterization

## Abstract

**Background:**

Pathogenic variants of the lysine acetyltransferase 6A or *KAT6A* gene are associated with a newly identified neurodevelopmental disorder characterized mainly by intellectual disability of variable severity and speech delay, hypotonia, and heart and eye malformations. Although loss of function (LoF) mutations were initially reported as causing this disorder, missense mutations, to date always involving serine residues, have recently been associated with a form of the disorder without cardiac involvement.

**Results:**

In this study we present five new patients, four with truncating mutations and one with a missense change and the only one not presenting with cardiac anomalies. The missense change [p.(Gly359Ser)], also predicted to affect splicing by in silico tools, was functionally tested in the patient’s lymphocyte RNA revealing a splicing effect for this allele that would lead to a frameshift and premature truncation.

**Conclusions:**

An extensive revision of the clinical features of these five patients revealed high concordance with the 80 cases previously reported, including developmental delay with speech delay, feeding difficulties, hypotonia, a high bulbous nose, and recurrent infections. Other features present in some of these five patients, such as cryptorchidism in males, syndactyly, and trigonocephaly, expand the clinical spectrum of this syndrome.

## Background

The lysine acetyltransferase 6A or *KAT6A* gene (a.k.a. *MYST3* and *MOZ*; MIM *601408) codes for a member of the histone acetyltransferase (HAT) family MYST. This gene was identified at a recurrent break-point of chromosomal translocations associated with acute myeloid leukaemia (AML) [1]. KAT6A acetylates lysine-9 residues in histone H3 (H3K9), playing an essential role in the regulation of transcriptional activity and gene expression. KAT6A is also involved in the acetylation and regulation of the tumor suppressor p53, a key factor in essential cell processes such as cell arrest and apoptosis [2]. Moreover, KAT6A is able to directly bind and regulate the transcription factors Runx1 and Runx2 through its C-terminal SM (serine and methionine rich) domain [3].

De novo mutations in *KAT6A* have recently been associated with a syndrome mainly characterized by intellectual disability (autosomal dominant mental retardation 32; MIM # 616268). To date, a total of 79 patients have been reported [4–15]. All of them present with developmental delay (DD) or intellectual disability (ID) with speech delay. Additionally, low muscle tone, problems with early feeding, and heart and eye defects are frequent [13]. Most of the reported mutations are loss of function (LoF) variants including splicing mutations, stop gain, and frameshift changes. Recently, missense mutations affecting highly conserved residues in relevant functional sites have also been described [12, 13].

The protein KAT6A is part of the MOZ/MORF complex together with ING5, KAT6B, MEAF6, and one of BRPF1–3. Neurodevelopmental syndromes have been associated not only with *KAT6A* but also with *KAT6B* and *BRPF1*. *KAT6B* mutations are associated with Say-Barber-Biesecker-Young-Simpson syndrome (SBBYSS, MIM # 603736) and Genitopatellar syndrome (MIM # 606170), while *BRPF1* mutations associate with IDDDFP (Intellectual developmental disorder with dysmorphic facies and ptosis, MIM # 617333). Like KAT6A syndrome, these two conditions present mainly with DD or ID and speech delay, hypotonia, and midline facial dysmorphic features including a broad nose.

Here, we present five new unrelated cases bearing four truncating mutations and one missense mutation in *KAT6A*, and we review the existing literature to expand the clinical delineation of KAT6A syndrome.

## Results

### Clinical description

The detailed phenotypic description of the patients is summarized in Table 1.

**Table 1:** Deep dysmorphological phenotyping after clinical evaluation of the 5 patients presented here

	Patient 1	Patient 2	Patient 3	Patient 4	Patient 5	Total
Variant’s genomic position	8:41792353	8:41792098	8:41792310	8:41834814	8:41791480	
cDNA change	c.3385C > T	c.3640A > T	c.3427_3428insTA	c.1075G > A	c.4254_4257 delTGAG	
Protein change	p.(Arg1129*)	p.(Lys1214*)	p.(Ser1143 Leufs*5)	p.Gly359Ser (p.Pro509 Thrfs*11)	p.(Glu1419 Trpfs*12)	
Exon	17/17	17/17	17/17	7/17	17/17	
Inheritance	De novo	De novo	De novo	De novo	De novo	5 de novo
dbSNP	rs786200960	–	–	–	–	
GnomAD	–	–	–	1/250564	–	
ClinVar	–	–	–	–	Pathogenic	
Current age (years)	16	11	9	8	6	10 av
Gender	Male	Male	Female	Male	Female	2F/3M
Ethnicity (country of origin)	Caucasian (Spain)	Caucasian (Spain)	Caucasian (Spain)	Caucasian (Spain)	Chinese (China)	
Neurological						
Global developmental delay (HP:0001263)/ Intellectual disability (HP:0001249)	+ (severe)	+	+	+	+	5/5
Autistic behaviour (HP:0007229)	NE	+	–	+	+	3/4
Speech delay (HP:0000750)	+	+	+	+	+	5/5
Seizures (HP:0001250)	+	+	–	+	–	3/5
Sleep disturbance (HP:0002360)	–	+	–	+	–	2/5
Hypotonia (HP:0001290)	–	–	–	+	+	2/5
Stereotypy (HP:0000733)	+	+	+	–	+	4/5
Lower limb hypertonia (HP:0006895)	+	+	+	–	–	3/5
Unstable gait (HP:0002141)	+	+	+	+	+	5/5
Craniofacial						
Microcephaly (HP:0000252)	+	+	+	+	+	5/5
Triangular face (HP:0000325)	+	+	+	–	+	4/5
Long face (HP:0000276)	+	–	+	+	+	4/5
Facial asymmetry (HP:0000324)	+	–	–	+ (mild)	–	2/5
Frontal bossing (HP:0002007)	+	–	–	+ (central)	–	2/5
Midface retrusion (HP:0011800)	–	+	+	–	+	3/5
Sparse medial eyebrows (HP:0025325)	+	+	+	+	+	5/5
Arched eyebrows (HP:0002553)	+	+	+	+	+	5/5
Thin eyebrows (HP:0045074)	+	+	+	–	–	3/5
Swollen skin on the upper eyelids (HP:0012724)	–	–	–	+	–	1/5
Epicanthal folds (HP:0000286)	+ (mild)	–	–	+	+	3/5
Proptosis (HP:0000520)	+	–	+ (mild)	–	–	2/5
Deep set eyes (HP:0000490)	–	–	+	+	–	2/5
High nasal bridge (HP:0000426)	+	–	–	–	+	2/5
Broad nasal tip (HP:0000455)	+	+	+	–	+	4/5
Bifid nasal tip (HP:0000456)	+	+	+	–	–	3/5
Prominent columella (HP:0009765)	+	–	–	–	–	1/5
Low-set ears (HP:0000369)	+	+	+	–	+	4/5
Anteverted ears (HP:0040080)	–	–	–	+	+	2/5
Prominent antihelix (HP:0000395)	+	+	+	+	+	5/5
Prominent antitragus (HP:0008593)	–	–	+	+ (mild)	+	3/5
Hypoplastic tragus (HP:0011272)	–	+	–	+	–	2/5
Small earlobe (HP:0000385)	+	–	+ (mild)	+ (mild)	–	3/5
Short philtrum (HP:0000322)	–	–	+	+ (mild)	–	2/5
Small mouth (HP:0000160)	+	–	–	–	+	2/5
Wide mouth (HP:0000154)	–	–	+	+	–	2/5
Prognathism (HP:0000303)	–	+	+	–	–	2/5
Pointed chin (HP:0000307)	+	+ (mild)	+	–	–	3/5
Ocular						
Convergent strabismus (HP:0000565)	+ (left eye)	+	+ (left eye)	–	+	4/5
Astigmatism (HP:0000483)	**–**	+	+	–	–	2/5
Myopia (HP:0000545)	+	+	–	–	–	2/5
Amblyopia (HP:0000646)	**+**	–	+	–	–	2/5
Nasolacrimal stenosis (HP:0000579)	**+**	–	+	–	–	2/5
Conjunctivitis (HP:0000509)	**–**	–	+	–	–	1/5
Thorax & Abdomen						
Long thorax (HP:0100818)	+	+	+	+	+	5/5
Narrow thorax (HP:0000774)	+	+	+	+	+	5/5
Asymmetric chest (HP:0001555)	pectus excavatum	–	–	–	–	0/5
Wide intermamillary distance (HP:0006610)	+	+	+ (mild)	+ (mild)	+	5/5
Low-set nipples (HP:0002562)	–	+	NE	+ (mild)	+	3/4
Inverted nipple (HP:0003186)	+ (left)	–	NE	–	–	1/4
Bulging abdomen (HP:0001538)	–	–	+	–	+	2/5
Prominent umbilicus (HP:0001544)	–	–	+	–	–	1/5
Limbs						
Skin syndactyly between 3rd and 4th fingers (HP:0011939)	+ (mild)	–	–	–	–	1/5
Upper limb amyotrophy (HP:0009129)	+	+	+	+	–	4/5
Lower limb amyotrophy (HP:0007210)	+	+	+	+	–	4/5
Lower limb asymmetry (HP:0100559)	–	–	+	NE	–	1/4
Genu valgum (HP:0002857)	+	–	+ (mild)	–	–	2/5
Genu varum (HP:0002970)	–	–	–	+ (mild)	–	1/5
Enlargement of the proximal interphalangeal joints (HP:0006185)	+	+ (mild)	–	–	–	2/5
Pes planus (HP:0001763)	+	+	+	+	–	4/5
Deviation of the hallux (HP:0010051)	+	–	+	–	–	2/5
Short halluces (HP:0010109)	–	–	+ (mild)	+	–	2/5
Sandal gap (HP:0001852)	–	–	–	+	–	1/5
Hammertoe (HP:0001765)	–	–	–	+	–	1/5
Other						
Prenatal problems (HP:0001197)	IUGR	–	Mild pyelectasis in left kidney; IUGR	–	–	2/5
Cardiovascular problems (HP:0001626)	+	+ (atrial septal defect)	+ (pulmonary stenosis)	–	+ (atrial septal defect)	4/5
Respiratory problems (HP:0002795)	–	+	–	–	–	1/5
Genitourinary problems (HP:0000119)	Small testis and penis; No sphincter control	–	–	Abnomal sphincter control	–	2/5
Feeding problems (HP:0011968)	+	+	+	+	+	5/5
Freckling (HP:0001480)	Freckles on the thorax	Numerous freckles (face and body)	–	–	–	2/5
Webbed neck (HP:0000465)	–	–	–	–	–	0/5

*Av* average, *IUGR* Intrauterine growth retardation. *NE* Not Evaluable

#### Patient 1 (P1)

The patient is a 10-year-old boy from a non-consanguineous and healthy couple. Two younger siblings, a girl aged 5½ years and a boy aged 3, are healthy. During the pregnancy of the proband, intrauterine growth retardation was detected. He was born at 37 weeks by caesarean section with Apgar scores 9/10 at 1 and 5 min, respectively. At birth, weight was 2.04 kg (2nd percentile, − 2.04 SD), height 43 cm (<3rd percentile, − 2.91 SD), and cranial circumference 30 cm (<3rd percentile, − 3.77 SD). The infant presented dysmorphic features such a triangular facies, low-set ears, and short neck, in addition to cryptorchidism. Abdominal and cranial ultrasound studies were performed with no evidence of malformations. Serology for common neonatal infections (toxoplasma, rubella, cytomegalovirus, and herpes simplex) as well as brain computerized tomography (CT) and funduscopy were normal.

At the 5th month of life, a prominent metopic suture was observed. The helical CT confirmed craniosynostosis of the metopic suture and ossification of the anterior fontanelle. At this follow-up visit developmental delay and limb hypertonia were detected. The subject began to crawl at 4 years of age and made his first unassisted steps at age 5. At 9 years of age absence seizures were clinically suspected and he was treated with valproate, showing good response. At 11 years the treatment was discontinued as the patient did not present seizures anymore and EEG was normal. Brain MRI (magnetic resonance imaging) was always normal. He was also treated with botulinum toxin because of hypersalivation due to dysphagia. The boy was also followed-up for severe myopia and constipation. He had a non-symptomatic large atrial septal defect, ASD (ostium secundum type).

**Fig. 1:**
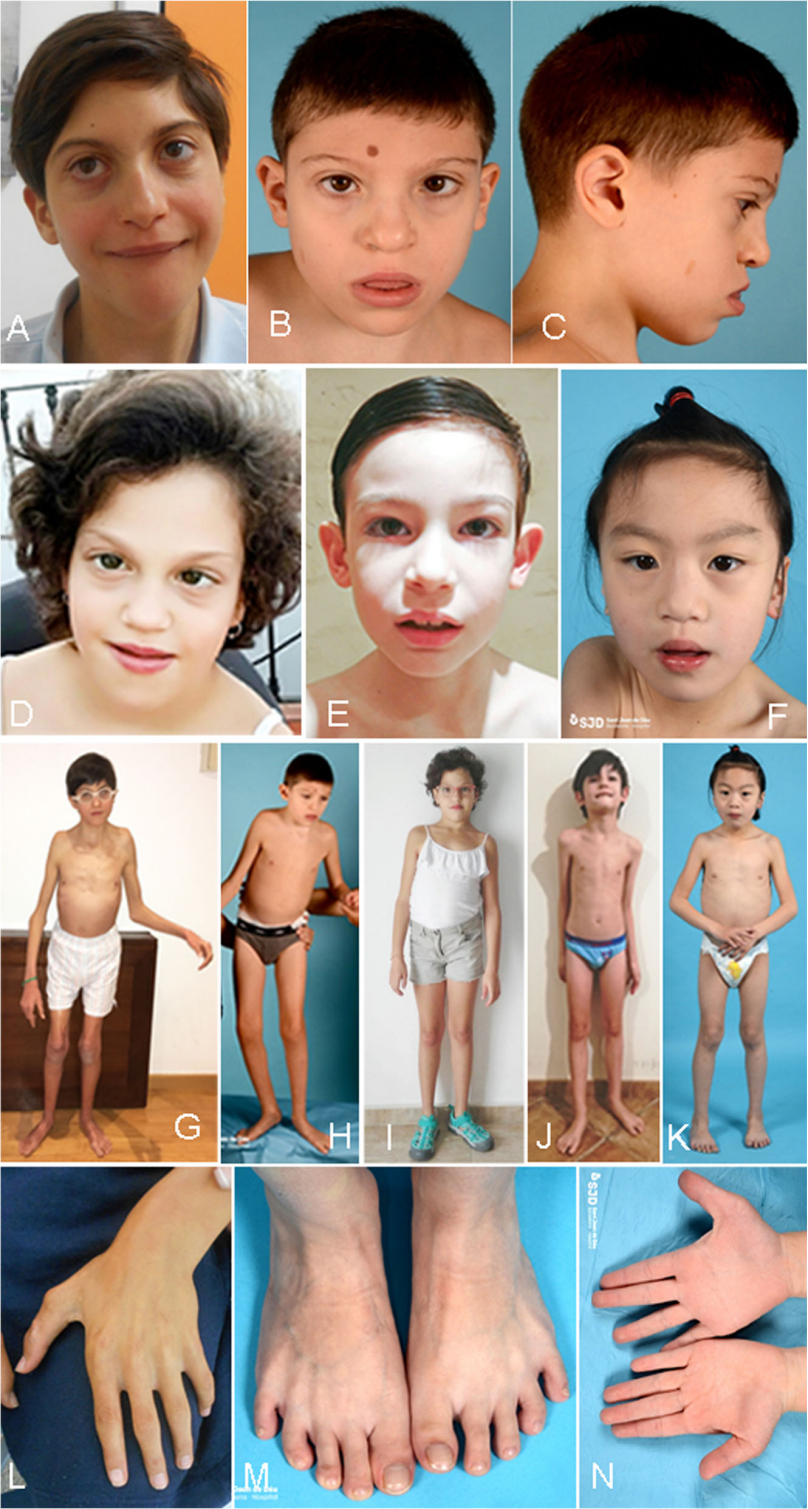
Images depicting key phenotypic features of the cases presented here. **a** Patient 1 facies at 16 years of age, **b** and **c** Patient 2 facies at 11 years, **d** Patient 3 facies at 9 years, **e** Patient 4 facies at 8 years, and **f** Patient 5 facies at 6 years. Panels **g** to **k** show the patient’s gestalt (at the same age as the facies figure). **l** Patient 1 hand. **m** and **n** Patient 5 ft and hands

At 10 years of age the dysmorphic traits continued, suggestive of Opitz C syndrome. The patient presented bulging eyes with true proptosis, mild epicanthus, hypoplastic nose, small mouth with normal philtrum and palate, and normal auricles (see detailed description in Table 1 and Fig. 1: a, g and l). From the neurological point of view he presented with spastic tetraparesis, with ulnar deviation of the hands, wide-base gait (increased distance between the tibial malleolus when walking) not in the range of a cerebellar ataxia but denoting clumsiness and a subtle gait disturbance, and flexion of hips and knees. The patient lacks expressive language, but has improved his ability to understand and to establish communication. He does not respond to simple commands and has poor attention span. He is unable to chew and is fed mashed food. He walks with increased lift base and can take some steps alone. Usually, he needs walker support to get around and has no sphincter control. The patient is currently on physiotherapy and speech therapy, and pharmacological treatment with risperidone and methylphenidate due to behavioural disturbances and ADHD (attention deficit-hyperactivity disorder) traits, respectively.

#### Patient 2 (P2)

The patient is an 11-year-old boy, the first child of healthy non-consanguineous parents. He was born at 38 + 5 weeks via caesarean section due to breech presentation after an uneventful pregnancy. At birth, weight was 2.97 kg (30th percentile, − 0.45 SD), length was 49 cm (25th percentile, − 0.28 SD), and cranial circumference was 33 cm (10th percentile, − 0.61 SD). On neonatal examination, sacral and suprasternal dimples were observed on ultrasound with normal subjacent tissues and reducible bilateral inguinal hernia.

The neonatal period was significant for poor feeding, mild hypertonia of limbs, and delayed gross motor development. He achieved head control at 3 months, sitting at 15 months, and crawling and kneeling later than 24 months; currently he walks only with a walker. His speech was also delayed as he started babbling at 22 months. His fine motor skills have improved with therapy; he is able to grab objects and play with both hands. Recurrent pneumonias were diagnosed with several hospital admissions, and dysphagia to liquids was observed, requiring thickeners. At age 2 years, an echocardiogram showed a 12-mm small ASD, ostium secundum type that was occluded with vascular plug system. Testing for inborn errors of metabolism (with plasma amino acids, acylcarnitine profile, total and free carnitines, and urine organic acids) was negative.

At age 6 years, he presented 2 episodes of loss of consciousness but electroencephalogram (EEG) did not confirm electric seizures. He was treated with valproic acid, currently on withdrawal. Brain MRI showed areas of polymicrogyria on the posterior right insula, delayed myelination, and slight descent of the cerebellar tonsils through the foramen magnum (Arnold-Chiari malformation).

On physical examination at age 11 years (Table 1 and Fig. 1: b, c and h), the patient was found to have a short stature (height 113 cm, <3rd percentile, − 4.75 SD), microcephaly (cranial circumference of 49.5 cm, <3rd percentile, − 3.74 SD), and dysmorphic facial features such as flat facies, hypertelorism, mildly blue sclera and proptosis, full lower lip and protruding tongue that led to an open mouth expression, and low-set dysplastic ears. Ocular abnormalities included endotropia, astigmatism, and myopia. Additionally, the patient had one right supernumerary nipple, a single palmar crease on the right hand, poor palmar sulcation, and mild fifth finger clinodactyly in both hands. Neurologically, he showed intellectual disability, poor eye contact, generalized hypertonia, the need for a stroller to walk, and absence of comprehensible language, but with babbling. He presents midline manual stereotypies and suffers sleep disturbances with frequent awakenings.

#### Patient 3 (P3)

The patient is a 9-year-old female, the only child of healthy non-consanguineous parents. Intrauterine growth retardation and mild left pyelectasis were detected during pregnancy. Birth weight was 2.30 kg (1st percentile, − 2.25 SD) and cranial circumference was 30.5 cm (<3rd percentile, − 3 SD). Screening for congenital disorders of metabolism was negative. Feeding difficulties and oral candidiasis were observed during the perinatal period. The subject achieved head control at 2 months, sat up at 10–12 months, began to crawl at 23 months, and presented unstable gait at age 3. At age 2, she was diagnosed with beta-thalassaemia minor (of paternal inheritance). Mild valvular pulmonary stenosis was also observed, although this problem had disappeared by 6 years of age. EEG was normal at the age of 3 years. Brain MRI, performed at few months of age, showed unspecific delayed myelination, although repeated imaging was considered normal when the patient was 3 years old.

At 6 years of age, when she was recruited by the SpainUDP (Spanish Undiagnosed Rare Diseases Program, http://spainudp.isciii.es/), she had global developmental delay, intellectual disability, language impairment, stereotypies, astigmatism, amblyopia, and recurrent conjunctivitis. Her physical exam showed craniofacial dysmorphisms including microcephaly, midface retraction, mild hypoplasia of the ear lobe, prominent antitragus, sparse medial eyebrows, proptosis, strabismus, broad and bifid nasal tip, short philtrum, prognathism, and big mouth (Table 1 and Fig. 1: d and i). She also had wide intermammillary distance, bulging abdomen, protruding umbilicus placed on a small depression of the abdomen, mild genu valgo, pes planus, and fibular deviation of halluces.

#### Patient 4 (P4)

The patient is an 8-year-old boy, the only child of non-consanguineous parents. No problems were detected during pregnancy and the neonatal period. He was born at term with a birth weight of 3.7 kg (76th percentile, + 0.70 SD) and a cranial circumference of 35 cm (34th percentile, − 0.42 SD). He achieved head control at 3 months, sat up at 8.5 months, and began to walk independently at 16 months. Microcephaly was detected at 2 months of age and he was diagnosed with atypical absences with eyelid myoclonias at the age of 16 months. He was treated with valproate from 21 months to 5 years with acceptable control of seizures. When the proband was 5 years old, it was withdrawn due to its association with adverse reactions (nauseas, vomiting, and weight loss). A few months later, valproate treatment was administered again since the number and intensity of epileptic crises had increased. In this recurrence of epilepsy, ethosuximide was needed together with valproate to achieve seizure control. Several EEGs carried out starting at age 3 years have disclosed spike-wave anomalies. When he was admitted to SpainUDP, at 6 years of age, the subject showed intellectual disability with altered fine motor coordination, unstable gait with frequent falls, language impairment, and autistic behaviour. He has always had sleep disturbances and hyporexia. Hypotonia with reduced muscle bulk has also been observed. Physical examination (Table 1 and Fig. 1: e and j) showed microcephaly, long face, mid facial asymmetry, frontal central bossing, swollen skin on the upper eyelids, epicanthus, deep set eyes, mildly everted lower eyelid in its external part, and deep horizontal groove under the lower lip. Additional features include a slender appearance, scarce body adiposity, long and narrow thorax, mild genu varo (bilateral), pes planus, short halluces, sandal gap between the first and second toes, and flexed fifth toes.

Metabolic testing (including urine organic acids and glucose in cerebrospinal fluid) was normal. Brain MRI was normal.

#### Patient 5 (P5)

The patient is a 6.5-year-old girl, first child of healthy non-consanguineous parents and with a healthy younger sister. The subject was born at term with an uneventful delivery and with a birth weight of 3.14 kg (42nd percentile, − 0.2 SD), a cranial circumference of 33 cm (10th percentile, − 0.61 SD), and Apgar 9/10 at 1 and 5 min. New-born screening for inborn errors of metabolism was normal. She had feeding problems from the neonatal period. During her hospital admission for dysphagia at 2 months of age, an atrial septal defect (ASD) was detected, which was surgically repaired at 16 months. She had delayed motor skills, with head control at 6 months, sitting up at 13 months, crawling at 16 months, and autonomous although unstable gait developing at age 2.

The subject has received physical, speech, and behavioral therapies and has attended a special school since 6 years of age. She is not able to talk, shows difficulties in adapting to new environments, shows significant behavioral problems with particular impact on the social domain, and has poor eye contact. Physical examination (Table 1 and Fig. 1: f, k, m and n) showed dysmorphic features characterized by midface hypoplasia, almond-shaped eyes, and slightly upslanted palpebral fissures, and absent Cupid’s bow. Ears have underdeveloped antihelix, and are slightly low set, with increased posterior angulation. The hands show a complex palmar dermatoglyphic pattern with abnormal square radial border morphology. Tone, strength, and deep tendon reflexes are normal.

Remarkably, cranial MRI at age 6 revealed prominent cerebellar interfolia space compared with a previous MRI at 2 years of age; this is compatible with progressive cerebellar atrophy. No other significant alterations were detected on MRI. The subjected was recruited by the Undiagnosed Rare Disease Program of Catalonia (www.urdcat.cat).

### Genetic results

#### Patient 1

A normal karyotype (46, XY) was determined. A trio-based whole exome study (WES) revealed a de novo heterozygous variant NM_006766:c.3385C > T (p.Arg1129*) in the *KAT6A* gene, which was confirmed by Sanger sequencing. This change was previously identified in a child with global developmental delay [5].

Other variants of unknown clinical relevance (VUS) are summarized in Additional file 1: Table S1.

#### Patient 2

Karyotype, subtelomeric fluorescent in situ hybridization (FISH), kit MLPA panel studying recurrent genomic disorders (SALSA® P245-B1), chromosomal microarray, and testing for fragile X, *MECP2*/*FOXG1* genes, Angelman syndrome, Pitt-Hopkins syndrome, mucopolysaccharidoses, and congenital disorders of glycosylation, yielded normal results. Clinical exome of the index case revealed a heterozygous de novo variant in *KAT6A* in the proband (NM_006766: c.3640 A > T (p.Lys1214*)]. Other VUS detected in the patient were: heterozygous c.852G > A (p.Leu284Leu) variant and heterozygous c.1467 + 16A > C variant in the *FOXG1* gene (Sup. Table 2).

**Table 2:** Clinical overview of KAT6A syndromic patients

	This report		Trinhet al. 2018 [12]	Efthymiou et al. 2018 [15]	Alkhateeb et al. 2019 [14]	Kennedy et al. 2019 [13]			Total				
Features	LT^a^	M^b^	M	LT	LT	ET	LT	M	ET	LT	M	Overall	%
Protein change	2FS; 2NS	p.G359S	p.N1975S	p.S1113*	p.K1130 fs*	10FS; 7NS; 1del	19FS; 29NS	6MS	10FS; 7NS; 1del	13FS; 38NS	8MS		
Gender (F/M)	2/2	0/1	1/1	0/1	0/1	8/10	25/23	2/4	8/10	27/27	3/6	38/43	
Perinatal features													
Small for gestational age	2/4	0/1	2/2	NR	0/1	2/15	8/44	0/4	2/15	10/49	2/7	14/71	20
Feeding difficulties/failure to thrive	4/4	1/1	1/1	1/1	1/1	10/18	40/46	4/6	10/18	46/52	6/8	62/78	**79**
Neonatal complications (low Apgar scores, espiratory distress …)	1/4	0/1	NR	0/1	0/1	0/1	4/5	NR	0/1	5/11	NR	5/12	42
Neurological features													
Global developmental delay/Intellectual disability	4/4	1/1	2/2	1/1	1/1	18/18	44/44	4/4	18/18	50/50	7/7	75/75	**100**
Speech delay/Absent speech	4/4	1/1	1/2	1/1	1/1	18/18	44/44	5/5	18/18	50/50	7/8	75/76	**99**
Unstable/abnormal gait	4/4	1/1	0/2	1/1	1/1		1/3	NR	0/0	7/9	1/3	8/12	67
Neonatal hypotonia	2/4	1/1	1/1	1/1	NR	8/18	40/47	5/6	8/18	43/52	7/8	58/78	74
Seizures	2/4	1/1	1/2	1/1	0/1	2/17	2/47	1/6	2/17	5/53	3/9	10/79	13
Sleep disturbance	1/4	1/1	NR	NR	NR	3/16	15/28	2/4	3/16	16/32	3/5	22/53	42
Autistic behavior/behavioral problems	2/3	1/1	NR	NR	NR	4/15	8/18	3/3	4/15	10/21	4/4	18/40	45
Craniofacial features													
Microcephaly	4/4	1/1	2/2	1/1	0/1	1/18	20/45	1/15	1/18	25/51	1/5	27/74	36
Frontal bossing/large forehead	1/4	1/1	NR	1/1	0/1	0/1	1/4	NR	0/1	3/10	1/1	4/12	33
Bitemporal narrowing	1/4	0/1	0/2	1/1	0/1	NR	3/3	NR	NR	5/9	0/3	5/12	42
Ear anomalies (large, low set, rotated, small earlobe …)	4/4	1/1	NR	1/1	NR	NR	9/11	0/1	NR	14/16	1/2	15/18	**83**
Palpebral ptosis	1/4	0/1	1/2	0/1	NR	3/18	7/45	0/6	3/18	8/50	1/9	12/77	16
Eye anomalies (proptosis, hypertelorism, deep set,)	4/4	1/1	2/2	1/1	1/1	NR	3/8	1/1	0/0	9/14	4/4	13/18	**72**
Epicanthal folds	2/4	1/1	0/2	NR	0/1	NR	2/7	0/1	0/0	4/12	1/4	5/16	31
Broad/bulbose nasal tip	4/4	0/1	2/2	1/1	0/1	16/18	35/40	3/5	16/18	40/46	5/8	61/72	**85**
Thin upper lip	0/4	0/1	2/2	0/1	1/1	7/17	28/38	2/4	7/17	29/44	4/7	40/68	59
Micrognathia	0/4	0/1	0/2	0/1	0/1	NR	6/12	0/1	0/0	6/18	0/4	6/22	27
Ocular problems													
Strabismus	4/4	0/1	2/2	NR	NR	9/17	27/47	1/5	9/17	31/51	3/8	43/76	57
Visual defects	4/4	0/1	2/2	NR	NR	9/17	26/38	1/3	9/17	30/42	3/6	42/65	**65**
Other features													
Congenital heart defect	4/4	0/1	0/2	NR	0/1	5/18	32/46	0/6	5/18	36/51	0/9	41/78	53
Reflux	2/4	0/1	NR	NR	NR	7/18	27/38	3/6	7/18	29/42	3/7	39/67	58
Constipation	1/4	0/1	1/2	NR	NR	4/16	18/28	3/6	4/16	19/32	4/9	27/57	47
Recurrent infections	1/4	0/1	NR	NR	NR	5/16	24/34	1/5	5/16	25/38	1/6	31/60	52

*AF* Anterior fontanelle, *CVI* Cortical visual impairment, *Del* Deletion, *ET* Early truncating, *FS* Frameshift, *GERD* Gastroesophageal reflux disease, *LT* Late truncating, *M* Missense, *NR* not reported, *NS* nonsense, *PDA* Patent ductus arteriosus; Features present in more than 60% of the patients are indicated in bold with % of the total

^a^ Patients 1–3 and 5. ^b^ Patient 4

#### Patient 3

Prior genetic and metabolic testing included karyotype, FISH at 4p16.3, array-CGH, and 7-dehydrocholesterol testing, all negative. Trio-based WES revealed a heterozygous de novo variant (NM_006766:c.3427_3428insTA) in *KAT6A* in the proband, which was confirmed through Sanger sequencing. This frameshift variant consists of an insertion of two nucleotides at exon 17 and it leads to the premature termination of the protein (p.Ser1143Leufs*5). The variant has not been previously described in the genomic databases.

#### Patient 4

Before WES, several genetic tests were carried out, with negative results: karyotype, array-CGH (60 K), Fragile X, Angelman, and diagnostic exome sequencing (DES) for epilepsy (543 genes). Trio-based WES was performed and a de novo *KAT6A* variant was identified in the patient (NM_006766:c.1075G > A), which was confirmed through Sanger sequencing. This variant predicts a replacement of guanine by adenine at codon 359 in exon 7 [p.(Gly359Ser)]. It has been found in only one carrier out of 250,564 alleles in gnomAD. It is predicted to be ‘deleterious’ or ‘putatively pathogenic’ by a variety of bioinformatic tools (PolyPhen2, Mutation Taster) and to be ‘tolerated’ by SIFT (score 0.28); CADD score is 16.9. Altogether, this variant is classified as ‘pathogenic’ according to the AMCG/AMP 2015 guidelines. In addition, the Human Splicing Finder software (3.1 version; January 10, 2018) predicts a potential alteration of splicing consisting of the generation of a cryptic acceptor site (AGTTCGAACT**A**GCC) at exon 7 (which was not experimentally observed, see below), and the loss of a predicted ESE (**G**GCCCTGG) for splicing factor SC35 (starting at c.1075G, which is not present in the mutant allele).

#### Patient 5

The subject has a normal karyotype (46,XX) and molecular karyotype by array-CGH (60 K). Singleton WES was performed followed by trio-based segregation with Sanger sequencing. The analysis showed a heterozygous de novo frameshift deletion of four nucleotides (NM_006766: c.4254_4257delTGAG) in exon 17 of the *KAT6A* gene, already defined as ‘pathogenic’ in ClinVar and reported by Kennedy et al. [13]. The mutant mRNA is predicted to be translated as a truncated protein with a premature termination 12 amino acids after the frameshift (p. Glu1419Trpfs*12). The variant has not been described in healthy population in the genomic databases.

### *KAT6A* expression analysis

**Fig. 2:**
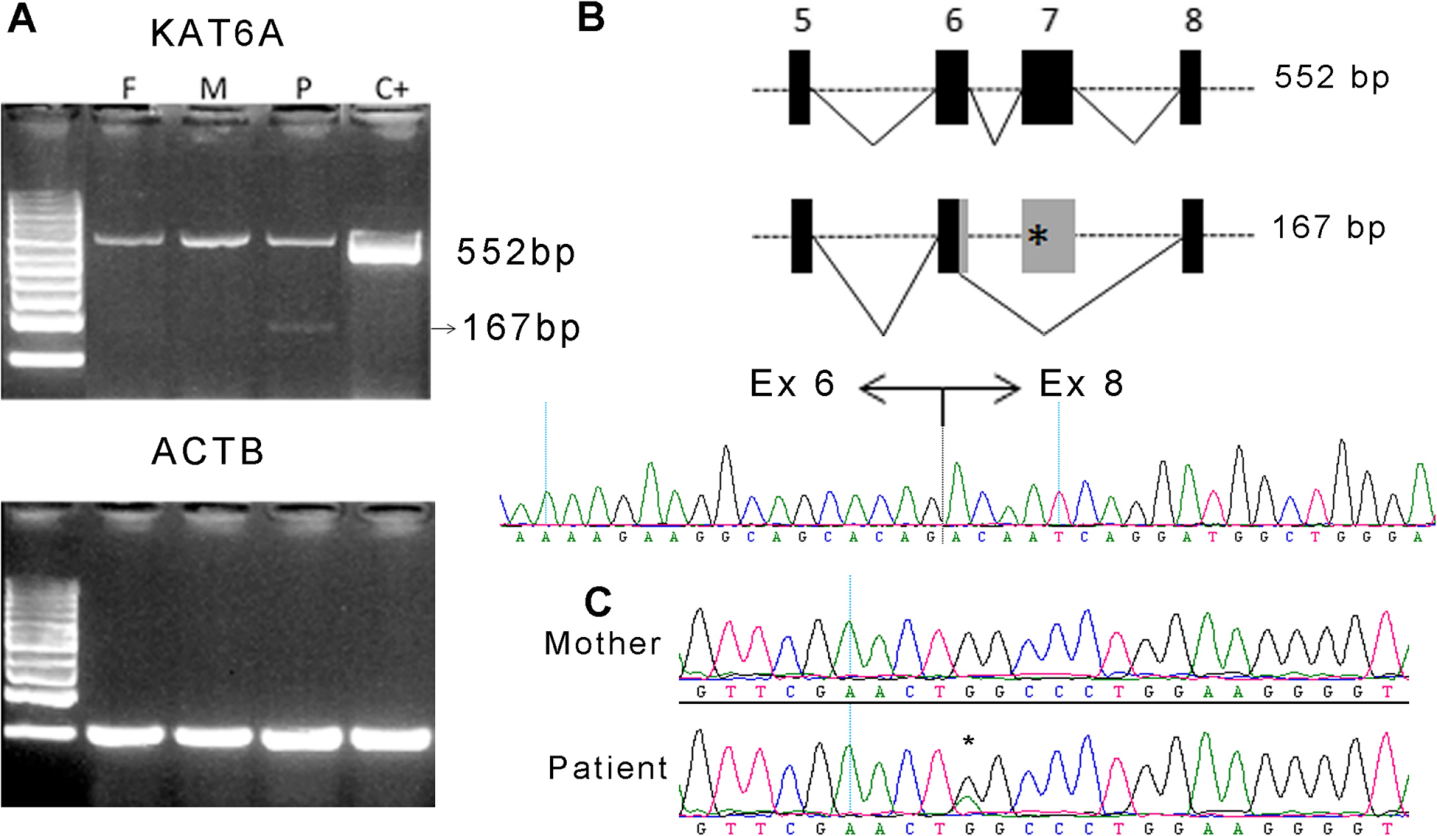
mRNA analysis of mutation c.1075G > A identified in Patient 4. **a** RT-PCR of *KAT6A* fragment including exon 5 to 8 in the patient (P), his mother (M) and father (F), and a control sample (C+). **b** Schematic representation of the normal (upper) and aberrant (lower) splicing of *KAT6A* exons 5 to 8 and chromatogram of exon 6–8 joining point in the smaller band of the patient. **c** Chromatograms of the mRNA amplification products in the mother and the patient upper band

Since pathogenic *KAT6A* mutations are usually truncating, and given that predictors indicate that the c.1075G > A variant identified in patient 4 may be affecting splicing mechanisms, we analysed the *KAT6A* splicing pattern of this patient using mRNA from peripheral blood cells. Expression analysis showed two bands: a normal amplicon of 552 bp and a shorter amplicon of 167 bp (with very low intensity) corresponding to an aberrant splicing (Fig. 2a). Sanger sequencing of this minor fragment revealed the loss of exon 7 (consistent with the predicted loss of an ESE), and of 65 additional bp of exon 6 (indicative of the use of a non-canonical cryptic donor site, Fig. 2b). The putative translation of this aberrant mRNA would involve a frameshift leading to a premature early termination codon (p.Arg330Serfs*13), potentially leading to nonsense mediated decay (NMD). The sequencing of the upper band showed both alleles (wild type and mutant), demonstrating that the allele carrying the missense mutation is mostly correctly spliced (Fig. 2c).

## Discussion

**Fig. 3:**
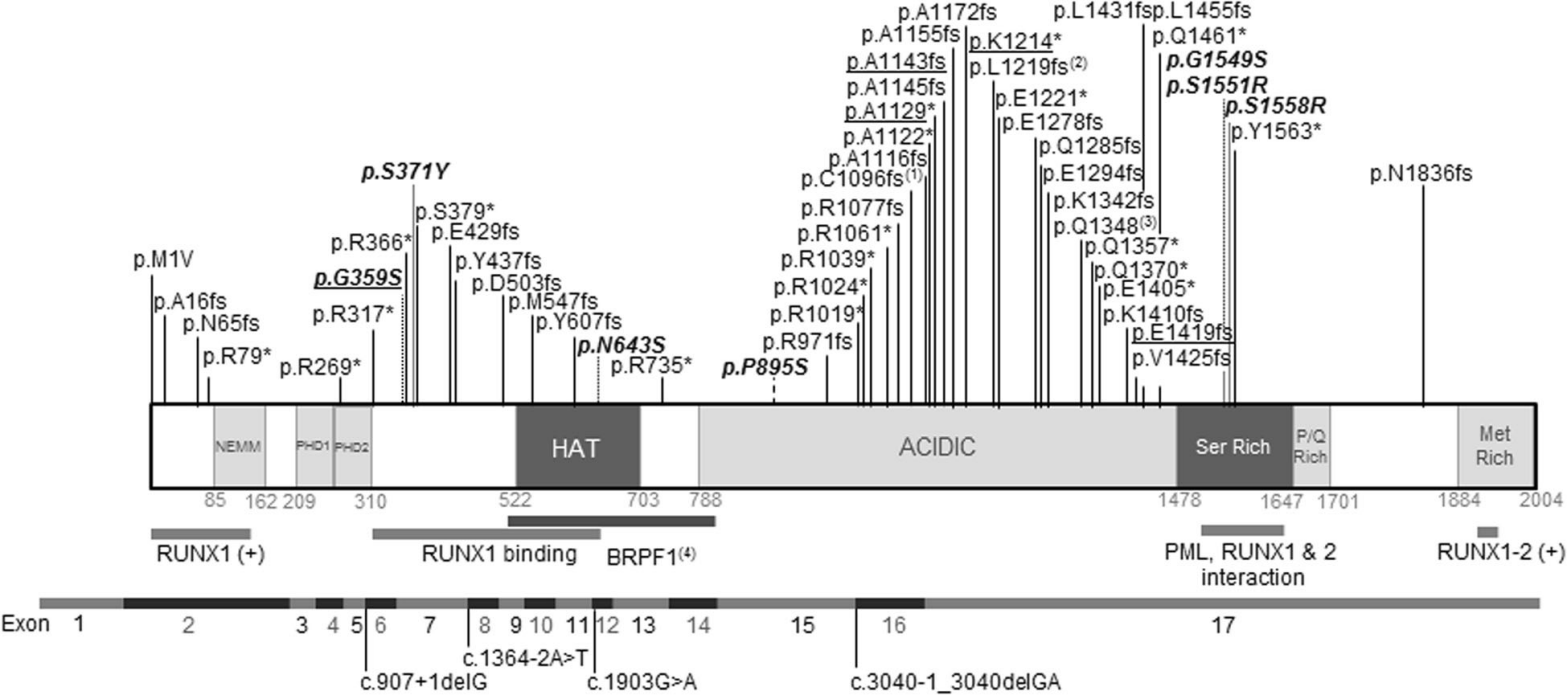
Schematic representation of KAT6A and localization of pathogenic variants, at the protein level (upper panel) and the exonic gene structure (scaled). (1) p.Cys1096Phefs*27; p.Cys1096Leufs*6; p.Cys1096Serfs*6; (2) p.Leu1219Thrfs*75; p.Leu1219Tyrfs*75; (3) p.Gln1348Argfs*7; p.Gln1348*; (4) Mediates interaction with BRPF1, required for histone H3 acetyltransferase activity; (+) activation. In bold, missense mutations. Underlined, mutations identified in the patients presented here

Since 2015, around 80 cases of syndromic intellectual disability due to mutations at the *KAT6A* gene have been described in the literature, delineating a new syndrome with variable presentation (Table 2 and Fig. 3) [4–15]. Here, we present 5 patients with de novo variants at *KAT6A*, four ‘late truncating’ and one missense variant, and we describe their clinical presentations, adding further clinical and molecular delineation to the KAT6A syndrome. The four late truncating mutations (in patients P1–3, and 5) are in the last exons and are thus predicted to escape NMD. The phenotypes of these patients are similar to those with late truncating mutations described by Kennedy et al. [13].

The main characteristic feature of this syndrome is neurological involvement. All five patients presented with moderate or severe developmental delay or intellectual disability with severe involvement of speech and expressive language being more affected than comprehension, as in nearly all KAT6A syndrome patients [13]. Also, all patients described here presented with motor delay, and two of them showed similar manual stereotypies (hand flapping or fluttering), a feature that has not been highlighted in previous reports. This finding combined with the poor eye contact and language impairment could represent part of the autistic spectrum behaviour, one of the most frequent and significant neurological symptoms. In addition, 3 out of the 5 patients presented here suffered epilepsy, a manifestation reported in only 13% of the previously described series.

While craniofacial dysmorphic features are present in all the patients, some of them are very unspecific and some patients present very mild alterations [12, 14], which renders clinical identification of this syndrome very challenging. Nevertheless, some facial similarities can be identified in several patients (such as P2 and P3). All five patients presented with microcephaly. Microcephaly has been reported in 36% of all patients, and nearly in half of the patients with late truncating mutations, but it is less frequent in patients bearing missense or early truncating mutations (Table 2). Also, patient 1 presented with trigonocephaly due to synostosis of the metopic suture. Different kinds of craniosynostoses have been previously reported [4], including sagittal synostosis [8, 13] and scaphocephaly [10]. Frontal bossing and/or bitemporal narrowing is reported in 30–40% of patients. In general, a broad/bulbous nasal tip is present in the majority of patients (86%) together with ocular anomalies (such as hypertelorism, proptosis or deep set eyes and downslanting palpebral fissures [6, 9, 12]), mouth anomalies (down-turned corners of the mouth [9] or protruding tongue [6, 7]) and ear anomalies (large, low set, rotated …), present in all five patients reported here. Joint hypermobility, a frequent finding in these five patients, has also been previously reported [9]. Other clinical findings previously reported are supernumerary nipple [6], cryptorchidism [6], and syndactyly [6].

Interestingly, all the present patients except for patient 4 (bearing a missense mutation) presented with congenital heart defects. Cardiac defects have been reported in around 70% of patients bearing late truncating mutations, but not hitherto in patients with missense mutations. Brain abnormalities, found in three of our patients (Patients 2, 3 and 5), have been frequently reported, including delayed myelination [13] and benign enlargement of the pericerebral areas [9], with the lack of the olfactory bulb [6] and pituitary abnormalities [11] being the two most consistent and noteworthy midline neuroimaging findings.

All five patients presented with feeding difficulties or failure to thrive, and patients 2, 4, and 5 also presented with neonatal hypotonia, traits reported in more than 70% of the *KAT6A* patients. Severe food allergies have been previously reported in 3 patients [8] and cow’s milk intolerance was noted in patient 42 in Kennedy et al. [13], although it is not clear if this is associated with mutations in *KAT6A*. None of the patients reported here presented with food intolerances or allergies.

Recurrent infections, observed in two patients of our cohort with late truncating variants, have been reported in nearly half of the previously published patients and in 71% of patients bearing late truncating variants [13]. This observation is in agreement with the improper B cell differentiation reported in the conditional KO murine model [16] and with the role of KAT6A as an essential factor for long-term repopulation of hematopoietic stem cells [17].

While the majority of *KAT6A* syndromic mutations are truncating, missense mutations have recently been described [12, 13]. Here we present a case with clinical characteristics similar to the rest of KAT6A patients, bearing the de novo missense mutation p.Gly359Ser. While *KAT6A* is clearly constrained against LoF variants (with a pLI = 1 and oe = 0.02, gnomAD accessed June 2019), it is not constrained for missense mutations, with an oe = 0.83, clearly above the recommended CI < 0.35. All the previous missense mutations associated with pathogenicity affect highly conserved residues in critical functional regions of the protein [13]. In addition, it is of note that all these changes involve serine residues, either eliminating an existing Ser or introducing a new one, as in the case of p.Gly359Ser, reported in patient 4. KAT6A C-terminal domain contains a serine- and methionine-rich domain that is essential for its binding to the transcription factor Runx2 [3]. As the hydroxyl group of serine is highly reactive and is able to form hydrogen bonds with a variety of polar substrates, the alteration of their number and position seems to be especially critical in KAT6A, otherwise tolerant of missense substitutions. In addition, we have verified that this variant can affect normal processing of pre-mRNA by producing an aberrant splicing consisting of the loss of exon 7 and 65 bp of exon 6. This mutation leads to early truncation and is assumed to be affected by the NMD process, reducing total *KAT6A* mRNA. It should be noted that this alteration seems to have a minor impact. It is likely that the pathological consequences of this variant are mainly due to the amino-acid substitution, leading to a deficit of functional KAT6A. Concordantly, the patient did not present with cardiac alterations, similar to the majority of patients bearing missense mutations.

## Conclusions

With this study, we have expanded the clinical delineation of KAT6A syndrome, an emerging and distinctive entity which is potentially clinically diagnosable. Given the severity of its clinical features and its reproductive implications, it is important to make an early diagnosis of this condition, including identification of those patients bearing missense mutations.

## Material and methods

### Biological samples

Genomic DNA of the patients and their parents was obtained from peripheral blood at the respective institutions (Hospital La Fe, Valencia, for P1, Hospital Sant Joan de Deu, Barcelona, for P2, Instituto de Salud Carlos III, Madrid, for P3 and P4, and Hospital del Mar, Barcelona, for P5). Signed informed consent was obtained from each patient’s parents. All protocols were approved by the ethics committee of each of the institutions and all methods were performed in accordance with the relevant guidelines and regulations.

### Whole exome sequencing and molecular analyses

#### Patient 1

Whole exome sequencing of the proband and his parents was performed in the National Centre of Genomic Analysis (CNAG; Barcelona, Spain) using the Illumina HiSeq-2000 platform. Exome capture was performed with Agilent SureSelect v5 (Agilent, CA, USA). The samples were sequenced at a coverage of 140x. The data were analyzed as described elsewhere [18]. Annotation, filtering, and prioritization of variants were carried out using VarAFT software [19]. The results were then filtered under de novo dominance and recessive hypotheses. Variants with a minimum allele frequency (MAF) above 0.001 for AD filtering and above 0.01 for AR filtering in the common population (according to GnomAD) were excluded. Variants in genes included in DDG2P (The Development Disorder Genotype-Phenotype Database [15, 20]), and covered by at least 10 reads, were prioritized for validation (it should be noted that those who carried out the original DECIPHER analysis and collection of the data bear no responsibility for the further analysis or interpretation of it).

The mean coverage was of 153.43, 174.29, and 160.829 reads for the patient, father and mother respectively, and 97.2–97.9% of the target region was covered with at least 10 reads (C10). A total of 5 variants in 4 genes were selected for validation by Sanger sequencing. Primer sequences and PCR conditions are available on request. PCR reaction, purification, and sequencing were performed as described previously [20].

#### Patient 2

Whole exome sequencing of the index case was carried out with the platform Illumina NextSeq500, using the Agilent SureSelect v6 QXT capture kit. For the analysis, several bioinformatics tools were used. The exome design covered approximately 95% of the coding regions of the analysed genes. The sequences used as reference can be found at the RefSeq database. The estimated frequencies were calculated from the 1000 genomes, Complete Genomics and NHLBI Exome Sequencing Project databases. The nomenclature of mutations was based on the recommendations of the Human Genome Variation Society. The analysis was carried out according to the recommendations of the American College of Medical Genetics. The results were then filtered under de novo dominance and recessive hypotheses. Variants with a minimum allele frequency (MAF) above 0.001 for AD filtering and above 0.01 for AR filtering in the common population (according to GnomAD) were excluded. Variants in genes included in DDG2P (The Development Disorder Genotype-Phenotype Database [21, 22]), and covered by at least 10 reads, were prioritized for validation (it should be noted that those who carried out the original DECIPHER analysis and collection of the data bear no responsibility for the further analysis or interpretation of it).

#### Patient 3

After exhaustive revision of clinical information of patient 3, she was admitted to the Spanish Undiagnosed Rare Diseases Program (SpainUDP) [23]. Also, this patient and her unaffected biological parents were enrolled in the FP7-funded ‘2016 BBMRI-LPC WES Call’ (Eurobiobank website, accessed on 19th July 2018) to carry out the research shown in this article.

Trio-based whole exome sequencing (WES) for this project was conducted at the Centro Nacional de Análisis Genómico (CNAG-CRG, Spain). SureSelect Human All Exon V5 (Agilent Technologies) was used to perform whole exome enrichment following the manufacturer’s instructions. The captured libraries were sequenced using TruSeq SBS Kit v3-HS (Illumina, Inc), in paired-end mode with a read length of 2x100bp. On average, 92x median coverage for each sample was generated in a fraction of a sequencing lane on HiSeq2000 following the manufacturer’s protocol. Images analysis, base calling, and quality scoring of the run were processed using the manufacturer’s software Real Time Analysis (RTA 1.13.48, HCS 1.5.15.1) and followed by generation of FASTQ sequence files by CASAVA. High-quality reads were aligned to the decoy version of the GRCh37 reference genome used by the 1000 Genomes Project (hs37d5) using BWA-MEM (version 0.7.8), and variants were identified following GATK Best Practices [24] using HaplotypeCaller (version 3.6). All variants with a minimum coverage of 8 reads and minimum genotype quality (GQ) of 20 were uploaded to the Genome-Phenome Analysis Platform (RD-Connect GPAP) [25, 26] for variant filtration and prioritization. Additionally, phenotypic terms were extracted from clinical documents stored in the patient registry by a member of SpainUDP, mapped to HPO (Human Phenotype Ontology) [27] terms, and uploaded to PhenoTips [28], a software tool available in the RD-Connect GPAP. This platform allows filtering and refining of the results by mode of inheritance, population frequencies, in silico pathogenicity prediction tools, and HPO codes [25, 26]. This filtering process was carried out by two independent researchers of SpainUDP with common criteria, and the results were compared in order to reach a consensus on selection of the candidate variants, which were confirmed by Sanger sequencing in all family members. Finally, various sources of information were consulted to build a report with a detailed review of the scientific evidence supporting the correlation between the detected causative variant and the proband’s phenotype.

#### Patient 4

Patient 4 was admitted to the SpainUDP and underwent phenotypic analysis following the standard criteria established by this program [23]. After peripheral blood genomic DNA isolation of patient 4 and his biological parents (see case 3 for details), trio-based WES and selection of candidate variants were performed as described by López et al. [23]. Variants assessed as pathogenic and possibly contributing to the proband’s phenotype were validated by Sanger sequencing in the full trio.

#### Patient 5

Patient 5 was admitted to the URDCat Program after deep phenotyping. Patient’s whole exome sequencing from extracted DNA from peripheral blood was carried out at the National Centre of Genomic Analysis (CNAG; Barcelona, Spain) using the Illumina HiSeq-2000 platform. Exome capture was performed with Nimblegen SeqCap EZ MedExome + mtDNA 47 Mb and the samples were sequenced at coverage of 90x. Sequencing data were analysed according to the project’s established pipeline, and afterwards SNV, indel, CNV, and mosaicism analysis was performed using the RDCat Genomic Analysis Platform. Variants assessed as pathogenic and possibly contributing to the proband’s phenotype were validated by Sanger sequencing in the trio.

### Gene expression analysis

In the case of patient 4, gene expression of *KAT6A* transcripts was evaluated in peripheral blood cells obtained from the proband and his progenitors. RNA was extracted from cells using RNeasy Mini Kit (Qiagen), and then reverse transcribed by Moloney Murine Leukemia Virus Reverse Transcriptase (M-MLV RT) (Promega), with 0.5 μg random hexamers (Thermofisher) and 1 μg total RNA. Reactions were incubated for 60 min at 37 °C in a thermocycler. Primer sequences for expression analysis were designed at exons 5 and 8, as follows: KAT6A_E5F: 5′-CCGAGGTTTTCACATGGAGT-3′ and KAT6A_E8R: 5′-CGCTCCTCATTTTCTTGTTTGC-3′. The ACTB gene was used as reference.

## Supplementary information

**Additional file 1: Table S1.** Variants of Unknown significance identified in Patient 1. **Table S2.** Variants of Unknown significance identified in Patient 2. **Table S3.** Variants of Unknown significance identified in Patient 3. **Table S4.** Variants of Unknown significance identified in Patient 4. **Table S5.** Variants of Unknown significance identified in Patient 5.

## Data Availability

The data that support the findings of this study are available on request from the corresponding author. The data are not publicly available due to privacy and ethical restrictions.
