# Supplementary material for: Five new cases of syndromic intellectual disability due to KAT6A mutations: widening the molecular and clinical spectrum
Source: Orphanet J Rare Dis. 2020 Feb 10;15:44. doi: 10.1186/s13023-020-1317-9 (PMC7011274; doi:10.1186/s13023-020-1317-9)
Supplement: Supplementary file 1 — Additional file 1: Table S1. Variants of Unknown significance identified in Patient 1. Table S2. Variants of Unknown significance identified in Patient 2. Table S3. Variants of Unknown significance identified in Patient 3. Table S4. Variants of Unknown significance identified in Patient 4. Table S5. Variants of Unknown significance identified in Patient 5. [file 13023_2020_1317_MOESM1_ESM.pdf]

Supplementary Table 1. Variants of Unknown significance identified in Patient 1.

Gene	cDNA	Protein	Zig	Disease[1] (MIM; Inh)	Origin	gnomAD MAF (Hom)	dbSNP	Effect (CADD/SIFT/ PP2)	Clin Var	Class[2] (ACMG)
ENPP1	c.350T>C	p.Phe117Ser	het	Cole disease (615522; AD)	De novo	-	-	24.8/T/D	-	Likely benign
APOB	c.3337G>C	p.Asp1113His	hom	Hypobetalipo-proteinemia (615558; AR)	Both parents	0.0069 (23)	rs12713844	24.1/D/D	Conflicting: B (7); LB(4); P(1); US(1)	Benign
RNF213	c.12817G>A	p.Asp4273Asn	het	*Susceptibility to Moyamoya disease 2 (607151)*	Pat	0. 0.0027 (2)	rs141329059	29.5/T/D	-	Likely benign
	c.14030G>T	p.Trp4677Leu	het		Mat	0.0104 (29)	rs61741961	28/D/D	-	Likely benign

Zig: homozygosity/heterozygosity; het: heterozygote; hom: homozygotes (number of homozigotes in GnomAD database); Inh: inheritance; Pat: paternal; Mat: maternal; SIFT (D - Damaging; T - Tolerated); PP2: Polyphen2 (D - Probably damaging; P - Possibly damaging; B - Benign).

1. Pathologies previously associated with mutations in these genes. In brackets MIM number of the associated disease and its inheritance (according to OMIM).
2. According with the ACMG/AMP 2015 guideline

Supplementary Table 2. Variants of Unknown significance identified in Patient 2.

Gene	c.DNA	Protein	Zig	Disease1 (MIM; Inh)	gnomAD MAF	dbSNP	Effect (CADD/SIFT/PP2)	Class[2] (ACMG)
MYOC	c.767C>T	p.Thr256Met	het	Glaucoma 1A, primary open angle (137750, AD)	0.000083	rs200072086	19,07/D/PD	LB
ITGA8	c.2445G>T	p.Glu815Asp	het	Renal hypodysplasia/aplasia 1 (191830, AR)	0.00048	rs112914197	10,95/T/B	LB
C2CD3	c.6833A>G	p.Asn2278Ser	het	Orofaciodigital syndrome XIV (615948, AR)	0.00038	rs199993353	23,2/D/B	US
UROC1	c.883C>T	p.Arg295Cys	het	Uncertain. Urocanase deficiency (276880, AR)	0.000134	rs372290750	33/D/PD	US
ARID1B	c.4234T>G	p.Ser1412Ala	het	Coffin-Siris syndrome 1 (135900, AD)	0.00012	rs145516400	8,831/T/B	LB
KCNQ3	c.2146G>C	p.Asp716His	het	Seizures, benign neonatal, 2 (121201, AD)	0.000055	rs149324120	16,04/T/B	LB

Zig: homozygosity/heterozygosity; het: heterozygote; Inh: inheritance; Pat: paternal; Mat: maternal; CADD (Phred-SCALED CADD score, indicating level of deleteriousness); SIFT (D - Damaging; T - Tolerated); PP2: Polyphen2 (D - Probably damaging; P - Possibly damaging; B - Benign); LB: Liekly Benign; US: Uncertain Significance

1. Pathologies previously associated with mutations in these genes. In brackets MIM number of the associated disease and its inheritance (according to OMIM). (*) Filtered variant in gnomAD

2. According with the ACMG/AMP 2015 guideline.

Note: none of these changes was found in ClinVar.

Supplementary Table 3. Variants of Unknown significance identified in Patient 3.

Gene	cDNA	Protein	Zig	Disease[1] (MIM; Inh)	Origin	gnomAD MAF	db SNP	Effect (CADD/SIFT/PP2)
TMPO	c.610G>T	p.Gly204Cys	het	-	De novo	-	-	21.4/D/D
APOBEC3F	c.340A>C	p.Thr114Pro	het	-	De novo	0.000164	rs753023597	20.8/D/D
PTPN22	c.2198C>A	p.Thr733Lys	het	Susceptibility to diabetes type 1 (222100; AR) & Susceptibility to rheumatoid arthritis (180300)	Pat	-	-	25.4/D/D
	c.1366C>G	p.Gln456Glu	het		Mat	0.001773	rs72650672	21.1/D/D
ERO1LB	c.278A>G	p.Lys93Arg	het	none	Mat	0.001224 (1)	rs147983087	< 20/T/B
	c.189A>C	p.Lys63Asn	het		Pat	0.002091 (4)	rs35648587	< 20/D/D
SYCP2L	c.177T>A	p.Asn59Lys	het	none	Pat	0.000004	rs1052522642	23.0/D/P
	c.212A>C	p.Asn71Thr	het		Pat	0.000004	rs780214095	< 20/T/P
	c.1100T>C	p.Ile367Thr	het		Mat	0.002272 (1)	rs148762988	23.2/D/D

Zig: homozygosity/heterozygosity; het: heterozygote; Inh: inheritance; Pat: paternal; Mat: maternal; CADD (Phred-SCALED CADD score, indicating level of deleteriousness); SIFT (D - Damaging; T - Tolerated); PP2: Polyphen2 (D - Probably damaging; P - Possibly damaging; B - Benign).

1. Pathologies previously associated with mutations in these genes. In brackets MIM number of the associated disease and its inheritance (according to OMIM).

Note: none of these changes was found in ClinVar.

Supplementary Table 4. Variants of Unknown significance identified in Patient 4.

Gene	cDNA	Protein	Zig	Disease[1] (MIM; Inh)	Origin	gnomAD MAF	dbSNP	Effect (CADD/SIFT/ PP2)	ClinVar classification
LRRC8D	c.1902G>A	p.Met634Ile	het	-	De novo	-	-	23.5/D/P	-
SLC4A8	c.1499C>G[7]	p.Ala500Gly	het	-	De novo	-	-	< 20/T/B	-
LTBP3	c.2856_2857 delCC	p.Asp952Glufs*41	het	Geleophysic dysplasia 3 (617809; AD)	De novo	-	-	.	-
SCFD2	c.848G>T	p.Gly283Val	hom	-	Pat/Mat	0.007204 (13)	rs79025139	28.7/D/D	-
GPR98	c.746G>A	p.Arg249Lys	het	Usher syndrome, type 2C (605472; AR)	Mat	0.006381 (15)	rs41303344	< 20/T/B	Conflicting: B(3);LB (2);LP (1)
	c.5830G>A	p.Asp1944Asn	het		Pat	0.004284 (6)	rs41302834	25.0/D/D	Benign/Likely benign

Zig: homozygosity/heterozygosity; het: heterozygote; hom: homozygote; Inh: inheritance; Pat: paternal; Mat: maternal; CADD (Phred-SCALED CADD score, indicating level of deleteriousness); SIFT (D - Damaging; T - Tolerated); PP2: Polyphen2 (D - Probably damaging; P - Possibly damaging; B - Benign).

1. Pathologies previously associated with mutations in these genes. In brackets MIM number of the associated disease and its inheritance (according to OMIM).

Supplementary Table 5. Variants of Unknown significance identified in Patient 5.

Gene	cDNA	Protein	Zig	Disease[1] (MIM; Inh)	Origin	gnomAD MAF	db SNP	Effect (CADD)
PC	c.2491G>A	p.Val831Met	het	Pyruvate carboxylase deficiency; (608786; AR)	NA	0.0000039	rs762323318	25.9
ATP1A2	c.1285G>A	p.Ala429Thr	het	Alternating hemiplegia of childhood; (104290; AD)	NA	0.00004 (5)	rs77608625	26.7
RENBP	c.1091A>C	p.Glu364Ala	het	-	NA	0.000006	-	26.8
PMPCA	c.1263+84delG	intron variant	het	Spinocerebellar ataxia, autosomal recessive 2 (213200; AR)	NA	0.000004	rs773313604	<20

Zig: homozygosity/heterozygosity; het: heterozygote; Inh: inheritance; CADD (Phred-SCALED CADD score, indicating level of deleteriousness). NA: Not Analyzed.
1. Pathologies previously associated with mutations in these genes. In brackets MIM number of the associated disease and its inheritance (according to OMIM)
Note: none of these changes was found in ClinVar.
